# The β and γ subunits play distinct functional roles in the α_2_βγ heterotetramer of human NAD-dependent isocitrate dehydrogenase

**DOI:** 10.1038/srep41882

**Published:** 2017-01-31

**Authors:** Tengfei Ma, Yingjie Peng, Wei Huang, Yabing Liu, Jianping Ding

**Affiliations:** 1 National Center for Protein Science Shanghai, State Key Laboratory of Molecular Biology, Center for Excellence in Molecular Cell Science, Institute of Biochemistry and Cell Biology, Shanghai Institutes for Biological Sciences, Chinese Academy of Sciences, 320 Yueyang Road, Shanghai 200031, China; 2 School of Life Sciences, Shanghai University, 333 Nanchen Road, Shanghai 200444, China; 3 School of Life Science and Technology, ShanghaiTech University, 100 Haike Road, Shanghai 201210, China; 4 Shanghai Science Research Center, Chinese Academy of Sciences, 333 Haike Road, Shanghai 201210, China; 5 Collaborative Innovation Center for Genetics and Development, Fudan University, 2005 Songhu Road, Shanghai 200438, China

## Abstract

Human NAD-dependent isocitrate dehydrogenase existing as the α_2_βγ heterotetramer, catalyzes the decarboxylation of isocitrate into α-ketoglutarate in the Krebs cycle, and is allosterically regulated by citrate, ADP and ATP. To explore the functional roles of the regulatory β and γ subunits, we systematically characterized the enzymatic properties of the holoenzyme and the composing αβ and αγ heterodimers in the absence and presence of regulators. The biochemical and mutagenesis data show that αβ and αγ alone have considerable basal activity but the full activity of α_2_βγ requires the assembly and cooperative function of both heterodimers. α_2_βγ and αγ can be activated by citrate or/and ADP, whereas αβ cannot. The binding of citrate or/and ADP decreases the *S*_0.5,isocitrate_ and thus enhances the catalytic efficiencies of the enzymes, and the two activators can act independently or synergistically. Moreover, ATP can activate α_2_βγ and αγ at low concentration and inhibit the enzymes at high concentration, but has only inhibitory effect on αβ. Furthermore, the allosteric activation of α_2_βγ is through the γ subunit not the β subunit. These results demonstrate that the γ subunit plays regulatory role to activate the holoenzyme, and the β subunit the structural role to facilitate the assembly of the holoenzyme.

Mitochondrial NAD-dependent isocitrate dehydrogenases (NAD-IDHs, EC 1.1.1.41) in eukaryotes are a family of enzymes that catalyze the oxidative decarboxylation of isocitrate (ICT) into α-ketoglutarate (α-KG) while reducing NAD to NADH in the rate-limiting step of the Krebs cycle. Those enzymes are conserved from yeast to mammals and consist of multiple subunits^1^. They are all allosteric enzymes under strict cellular regulation, which can be activated by AMP (in yeast) or ADP (in mammals) and citrate (CIT) but inhibited by ATP (in mammals) and NADH. Thus, the cellular ratios of [ATP]/[AMP or ADP] and [NADH]/[NAD] can regulate the activity of NAD-IDH in the Krebs cycle and influence the energy production and substance flux in cells.

Mammalian NAD-IDHs isolated from porcine heart^2^,^3^,^4^,^5^,^6^,^7^,^8^, porcine liver^9^, bovine heart^10^,^11^,^12^, and ox brain^13^ have been biochemically characterized. All these enzymes are consisted of three types of subunits in the ratio of 2α:1β:1γ, and exist as the α_2_βγ heterotetramer composed of the αβ and αγ heterodimers, which can be further dimerized into a heterooctamer (the heterotetramer and heterooctamer are sometimes called holoenzyme and hereafter we will not distinguish the heterotetramer, heterooctamer and holoenzyme in the biochemical context unless otherwise specified). The α, β and γ subunits have molecular masses of 37 kDa, 39 kDa and 39 kDa, respectively, and exhibit distinct isoelectric points. The α and β subunits share about 40% sequence identity, the α and γ subunits about 42% sequence identity, and the β and γ subunits about 52% sequence identity^14^,^15^. The previous biochemical studies showed that the individual α, β or γ subunit of porcine heart NAD-IDH isolated using urea (2 M) is either inactive or exhibits very low activity, but the recombined α and β subunits or α and γ subunits in the form of the αβ or αγ heterodimer shows considerable activity^2^. All mammalian NAD-IDHs require divalent metal ions such as Mn^2 + ^, Mg^2 + ^, Co^2 + ^, and Cd^2 + ^for the activity^16^,^17^,^18^,^19^. In addition, the activity could be positively regulated by CIT and ADP but inhibited by ATP and NADH^7^,^11^,^20^. It was suggested that these enzymes have two binding sites per heterotetramer for each ligand, including ICT, Mn^2 + ^, NAD, and ADP^4^,^21^.

Recombinant human NAD-IDH was successfully expressed in *E. coli*, which allowed more mutagenesis and biochemical studies of the enzyme based on sequence alignment with *E. coli* and porcine mitochondrial NADP-IDHs^19^,^22^,^23^,^24^,^25^. Those studies examined the functional roles of several strictly conserved residues of the α, β and γ subunits in the bindings of metal ion, ICT, NAD, and ADP, and showed that the α subunit is critical for the catalytic activity, and the β and γ subunits play functional roles in allosteric regulation; however, the α subunit alone has no detectable activity and its coexistence with one of the two regulatory subunits is essential for the activity.

*Saccharomyces cerevisiae* NAD-IDH exists as a heterotetramer composed of two heterodimers of the regulatory subunit IDH1 and catalytic subunit IDH2, which can be further assembled into a heterooctamer. The structures of yeast NAD-IDH in apo form, in CIT bound form, and in CIT-AMP bound form have been determined at moderate resolution^26^, which revealed the assembly of the heterotetramer and heterooctamer and the binding sites of the activators. IDH1 and IDH2 form a compact heterodimer that acts as the basic functional and structural unit, and the structural communication between the two subunits is considered to be important to the allosteric regulation.

As human NAD-IDH contains three types of subunits, the molecular mechanism of allosteric regulation appears to be more complex than that of yeast NAD-IDH. In addition, the enzymatic properties of the composing αβ and αγ heterodimers and the exact functional roles of the β and γ subunits in the α_2_βγ heterotetramer are not well understood. Thus, human NAD-IDH is a very good model for studying the molecular mechanism of allosteric regulation. Moreover, mutations of human NADP-dependent IDHs localized in the cytoplasm and mitochondria (also called IDH1 and IDH2) have been identified in multiple types of tumors and the mutant proteins confer neomorphic activity to convert α-KG into 2-hydroxyglutarate (2-HG) whose accumulation can result in epigenetic dysregulation, leading to oncogenesis and development of tumors^27^,^28^,^29^,^30^,^31^,^32^. Intriguingly, human NAD-IDH (also called IDH3) has also recently been implicated in some diseases. Homozygous mutations of the β subunit are suggested to be a cause of retinitis pigmentosa, a hereditary degeneration of the retina leading to blindness^33^. Aberrant expression of the α subunit can promote malignant tumor growth by inducing HIF-1-mediated metabolic reprogramming and angiogenesis^34^. Abnormalities of human NAD-IDH are associated with pathogenesis of major psychiatric disorders^35^. Furthermore, human NAD-IDH is shown to be a novel target of tributyltin, an environmental contaminant chemical, in human embryonic carcinoma cells^36^. Thus, the structural and mechanistic studies of human NAD-IDH have also important biomedical implications.

In this work, we systematically studied the enzymatic properties the αβ and αγ heterodimers and the α_2_βγ heterotetramer of human NAD-IDH in the absence and presence of several regulators including CIT, ADP and ATP. Our biochemical data show that the αγ heterodimer has similar enzymatic properties and kinetic parameters as the α_2_βγ heterotetramer, whereas the αβ heterodimer has only basal activity and cannot be allosterically regulated. The catalytic efficiencies of the α_2_βγ and αγ enzymes are activated by CIT or/and ADP through decreasing the *S*_0.5_ for ICT. Additionally, the two activators can function synergistically, suggesting that CIT and ADP can bind to the enzymes independently and cooperatively. Moreover, ATP can activate the α_2_βγ and αγ enzymes at low concentration in a manner similar to ADP and inhibit the activities at high concentration, but has only inhibitory effect on the αβ enzyme. Furthermore, the mutagenesis data show that the αβ and αγ heterodimers contribute equally to the activity of the α_2_βγ heterotetramer in either the absence or presence of the activators, and the allosteric regulation of the α_2_βγ heterotetramer is through the γ subunit not the β subunit. These results together demonstrate that the γ subunit plays the regulatory role to activate the holoenzyme, and the β subunit the structural role to facilitate the assembly of the holoenzyme. These findings provide new insights into the molecular mechanisms of the function and allosteric regulation of mammalian NAD-IDHs.

## Results

### Preparation of human NAD-IDH

**Figure 1:**
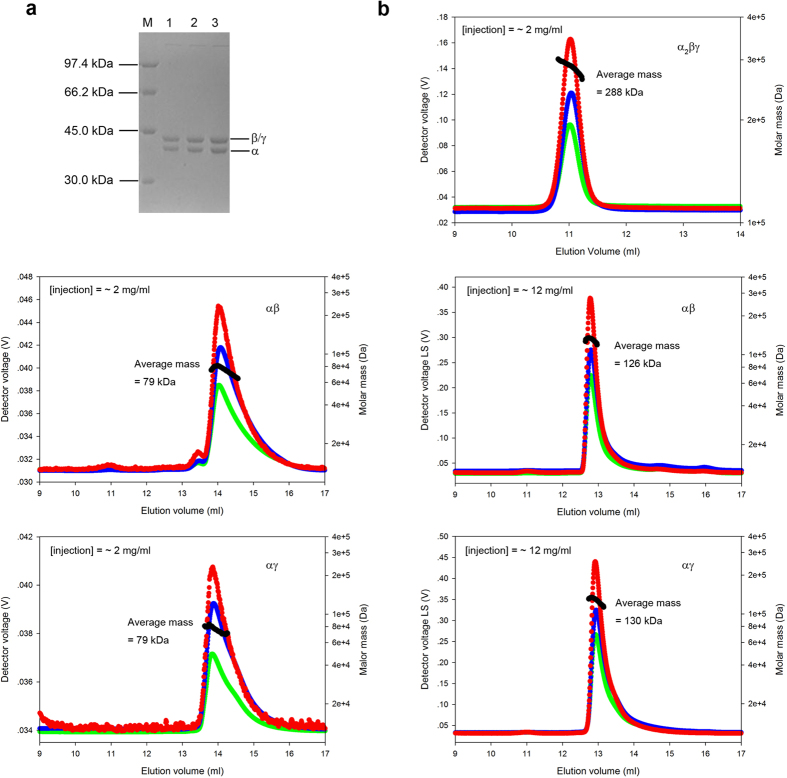
SDS-PAGE and SEC-MALS analyses of the α_2_βγ heterotetramer and the αβ and αγ heterodimers of human NAD-IDH. (**a**) SDS-PAGE (12%) analyses of the α_2_βγ heterotetramer and the αβ and αγ heterodimers with Coomassie blue staining. M, molecular mass standards; lane 1, the α_2_βγ heterotetramer; lane 2, the αβ heterodimer; lane 3, the αγ heterodimer. The upper band represents the β or/and γ subunits (39 kDa), and the lower band represents the α subunit (37 kDa). The ratio of the catalytic subunit and regulatory subunit(s) in all three samples is 1:1. (**b**) SEC-MALS analyses of the α_2_βγ, αβ and αγ proteins. Chromatograms show the readings from the light scattering (red) at 90°, refractive index (blue), and UV (green) detectors. The left and right vertical axes represent the light scattering detector reading and the molecular mass. The black curve represents the calculated molecular mass. The αβ and αγ proteins show an elution peak at about 14 ml corresponding to an average molecular mass of about 79 kDa at the injection protein concentration of 2 mg/ml, and an elution peak at about 13 ml corresponding to an average molecular mass of about 130 kDa at the injection protein concentration of 12 mg/ml. The α_2_βγ protein shows an elution peak at about 11 ml corresponding to an average molecular mass of about 288 kDa at the injection protein concentration of 2 mg/ml.

The αβ and αγ heterodimers and the α_2_βγ heterotetramer of human NAD-IDH were prepared as described in “Methods”. *In vitro* assembly of the α_2_βγ heterotetramer from the αβ and αγ heterodimers gave a much higher yield than the co-expression method, and the α_2_βγ heterotetramer obtained using both methods exhibited no difference in enzymatic properties, and thus we used the assembled α_2_βγ heterotetramer in all the biochemical studies. SDS-PAGE analyses showed that the purified α_2_βγ protein and αβ and αγ proteins are of sufficient purity (>95%) and there are two protein bands with equal intensity that are characteristic of mammalian NAD-IDHs^5^,^25^,^37^, with the upper band corresponding to the β or/and γ subunits (39 kDa) and the lower band corresponding to the α subunit (37 kDa) (Fig. 1a). Size-exclusion chromatography coupled with multi-angle light scattering (SEC-MALS) analyses showed that the α_2_βγ protein exhibits an elution volume of about 11 ml with an average molecular mass of about 288 kDa corresponding to a dimer of heterotetramer or a heterooctamer (theoretical molecular mass of 304 kDa), which is in agreement with the previous reports for the recombinant human NAD-IDH^24^,^25^ and the isolated pig heart NAD-IDH^3^. Both the αβ and αγ proteins exhibit an elution volume of about 14 ml at the injection protein concentration of 2 mg/ml with an average molecular mass of about 79 kDa corresponding to the αβ and αγ heterodimers (theoretical molecular mass of 76 kDa), but exhibit an elution volume of about 13 ml at the injection protein concentration of 12 mg/ml with an average molecular mass of about 130 kDa corresponding to dimers of heterodimers (theoretical molecular mass of 152 kDa) (Fig. 1b). These results indicate that the α_2_βγ protein exists as a heterooctamer in solution; the αβ and αγ proteins exist as heterodimers at low concentration but as dimers of heterodimers at high concentration. As the final protein concentrations in the enzymatic studies were below 0.2 μg/ml, presumably the αβ and αγ proteins exist mainly as heterodimers and the α_2_βγ protein as a heterooctamer.

### Enzymatic properties of human NAD-IDH

The α_2_βγ protein has a specific activity of 20.2 ± 0.3 μmol/min/mg at the standard conditions, and the αβ and αγ proteins exhibit specific activities of 3.33 ± 0.13 μmol/min/mg and 7.27 ± 0.31 μmol/min/mg (about 16.5% and 36.0% of that of the α_2_βγ protein), respectively, which are comparable to those of the isolated pig heart NAD-IDH^2^ and the purified α_2_βγ enzyme of human NAD-IDH^19^, but are significantly higher than those of the unpurified human NAD-IDH^22^ (Table 1). These results indicate that both the αβ and αγ heterodimers have considerable basal activities with the αγ heterodimer having about 2-fold higher activity than the αβ heterodimer, and the full activity of the α_2_βγ heterotetramer requires the assembly and cooperative functions of both heterodimers.

The previous biochemical data showed that mammalian NAD-IDHs require divalent metal ions for their activities^2^,^19^. Consistently, our biochemical data show that the α_2_βγ, αβ and αγ enzymes all require divalent metal ions for their activities but have different *S*_0.5_ values. The α_2_βγ enzyme has a *S*_0.5_ value of 60.2 ± 6.0 μM for Mn^2 + ^, the αγ enzyme has a slightly higher *S*_0.5_ (1.6-fold), but the αβ enzyme has a significantly higher *S*_0.5_ (88-fold) (Table 2). We also analyzed the effects of different metal ions on the activities of these enzymes, and among the six metal ions tested (Mn^2 + ^, Mg^2 + ^, Co^2 + ^, Zn^2 + ^, Ni^2 + ^and Ca^2 + ^), Mn^2 + ^is the most effective one (Supplementary Table S1). Thus, we used Mn^2 + ^in all the activity assays and kinetic studies.

### Activation of human NAD-IDH by CIT or/and ADP

The previous biochemical data showed that the activities of mammalian NAD-IDHs can be positively regulated by CIT and ADP^1^,^11^,^20^. To investigate the activation effects of CIT or/and ADP on the α_2_βγ, αβ and αγ enzymes, we first measured their kinetic parameters in the absence of any regulators. Our results show that the α_2_βγ enzyme has a *V*_max,ICT_ of 20.0 ± 0.1 μmol/min/mg, and *S*_0.5,ICT_, *S*_0.5,Mn_ and *S*_0.5,NAD_ of 2.35 ± 0.05 mM, 60.2 ± 6.0 μM, and 143 ± 5 μM, respectively (Table 2). The αγ enzyme has a *V*_max,ICT_ of 7.29 ± 0.11 μmol/min/mg and *S*_0.5,ICT_, *S*_0.5,Mn_ and *S*_0.5,NAD_ of 4.49 ± 0.15 mM, 95.1 ± 3.2 μM, and 238 ± 18 μM, respectively, which are comparable to those of the α_2_βγ enzyme. As a result, the basal catalytic efficiency (*k*_*cat*_*/S*_0.5,ICT_) of the αγ enzyme is about 19.0% of that of the α_2_βγ enzyme. As the αβ enzyme has a very high *S*_0.5,Mn_ and its kinetic parameters could not be obtained at the standard conditions, we measured its kinetic parameters at a substantially increased concentration of MnCl_2_ (50 mM *vs*. 2 mM). At this condition, the αβ enzyme has a slightly lower *V*_max,ICT_ (10.9 ± 0.3 μmol/min/mg) but moderately to substantially higher *S*_0.5,ICT_ (13.4 ± 0.1 mM, 5.7-fold), *S*_0.5,Mn_ (5305 ± 314 μM, 88-fold) and *S*_0.5,NAD_ (326 ± 15 μM, 2.3-fold) than the α_2_βγ enzyme, and the basal catalytic efficiency is about 9.5% of that of the α_2_βγ enzyme (Table 2). These data indicate that in the absence of any regulators, the αγ enzyme exhibits similar kinetic properties and has comparable kinetic parameters as the α_2_βγ enzyme, but the αβ enzyme does not.

**Figure 2:**
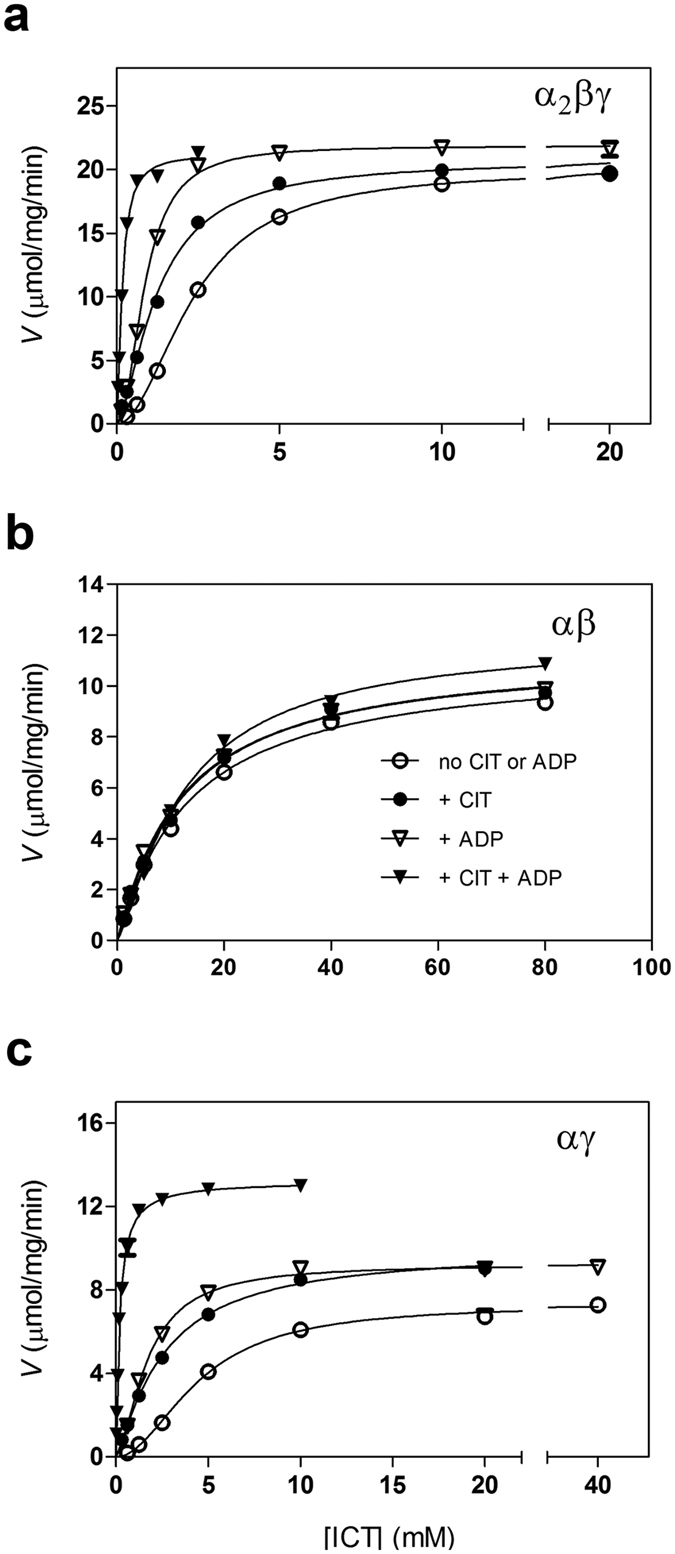
Saturation curves of the α_2_βγ heterotetramer and the αβ and αγ heterodimers for isocitrate in the absence and presence of positive regulator(s). (**a**) Saturation curves of the α_2_βγ enzyme. (**b**) Saturation curves of the αβ enzyme. (**c**) Saturation curves of the αγ enzyme. The activities of the α_2_βγ and αγ enzymes were measured at the standard conditions (33 mM Tris-acetate, pH 7.4, 2 mM MnCl_2_, and 3.2 mM NAD) with varied concentration of ICT in the absence or presence of 1 mM CIT or/and 1 mM ADP. The activity of the αβ enzyme was measured at the standard conditions but with higher concentration of Mn^2 + ^(50 mM). The derived *V*_max_, *S*_0.5_ and *k*_*cat*_ are listed in Tables 2 and 3.

We then measured the kinetic parameters of the α_2_βγ, αβ and αγ enzymes in the presence of activator(s) (Fig. 2 and Table 3). Compared with those in the absence of any regulators, in the presence of CIT, ADP and both activators, the *V*_max,ICT_ of the α_2_βγ enzyme has no significant change but the *S*_0.5,ICT_ is decreased by 1.9, 2.7 and 14.6 folds, respectively; and similarly, the *V*_max,ICT_ of the αγ enzyme is slightly increased by 1.2–1.8 folds but the *S*_0.5,ICT_ is decreased by 1.7, 2.7 and 24.7 folds, respectively. In contrast, addition of CIT or/and ADP has no significant effects on the *V*_max,ICT_ and *S*_0.5,ICT_ of the αβ enzyme. As a result, the catalytic efficiencies of the α_2_βγ and αγ enzymes are substantially increased by about 15.3 and 44.8 folds, respectively; whereas that of the αβ enzyme is not affected. Thus, the catalytic efficiency of the αγ enzyme is elevated to about 55.6% of that of the α_2_βγ enzyme, while that of the αβ enzyme is only about 0.7% of that of the α_2_βγ enzyme (Table 3). These data indicate that the binding of CIT or/and ADP to the α_2_βγ and αγ enzymes potentiates their activities mainly through decreasing the *S*_0.5,ICT_ without significantly affecting the *V*_max_ and the *k*_cat_. As either CIT or ADP can slightly decrease the *S*_0.5,ICT_ of the α_2_βγ and αγ enzymes but the two activators together can substantially decrease the *S*_0.5,ICT_ (Table 3), these data suggest that the two activators can bind either independently or simultaneously to distinct sites at the allosteric site, and the two activators function in a synergistic manner. These results are consistent with the previous biochemical data showing that ADP can positively regulate the activity of mammalian NAD-IDH^7^, and the binding of ADP and CIT can lower the *S*_0.5,ICT_ but has no effect on the *V*_max_^20^,^38^. These data further demonstrate that in the presence of CIT or/and ADP, the αγ enzyme also exhibits similar kinetic properties as the α_2_βγ enzyme, but the αβ enzyme does not.

Moreover, our kinetic data show that in the absence of any regulators, both the α_2_βγ and αγ enzymes exhibit a Hill coefficient of 2 with respect to ICT, whereas the αβ enzyme exhibits a Hill coefficient of 1, indicating that the α_2_βγ and αγ enzymes contain at least two ICT-binding sites with positive cooperativity whereas the αβ enzyme contains only one ICT-binding site (Table 2). In the presence of CIT, ADP and both activators, the α_2_βγ enzyme still exhibits a Hill coefficient of 1.5, 2.0, and 1.5, respectively, indicating a cooperative binding for ICT in all these conditions; whereas the αγ enzyme exhibits a Hill coefficient of 1.2, 1.6, and 1.0, respectively, indicating that there is a cooperative binding for ICT only in the absence of CIT (Table 3). As expected, the Hill coefficient of the αβ enzyme is not affected by CIT or/and ADP. These results suggest that the αγ heterodimer may contain an ICT-binding site each in the α and γ subunits; CIT appears to bind to one site most likely in the γ subunit and thus eliminates the positive cooperativity, but the ADP binding does not occlude the binding of ICT to this site and thus retains the cooperativity. The αβ enzyme contains only one ICT-binding site very likely in the α subunit but no ICT-binding site in the β subunit. As the α_2_βγ enzyme exists as a dimer of heterotetramer, there is a positive cooperative binding for ICT between the two heterotetramers or/and the αβ and αγ heterodimers in both the absence and presence of regulators.

### Activation and inhibition of human NAD-IDH by ATP

**Figure 3:**
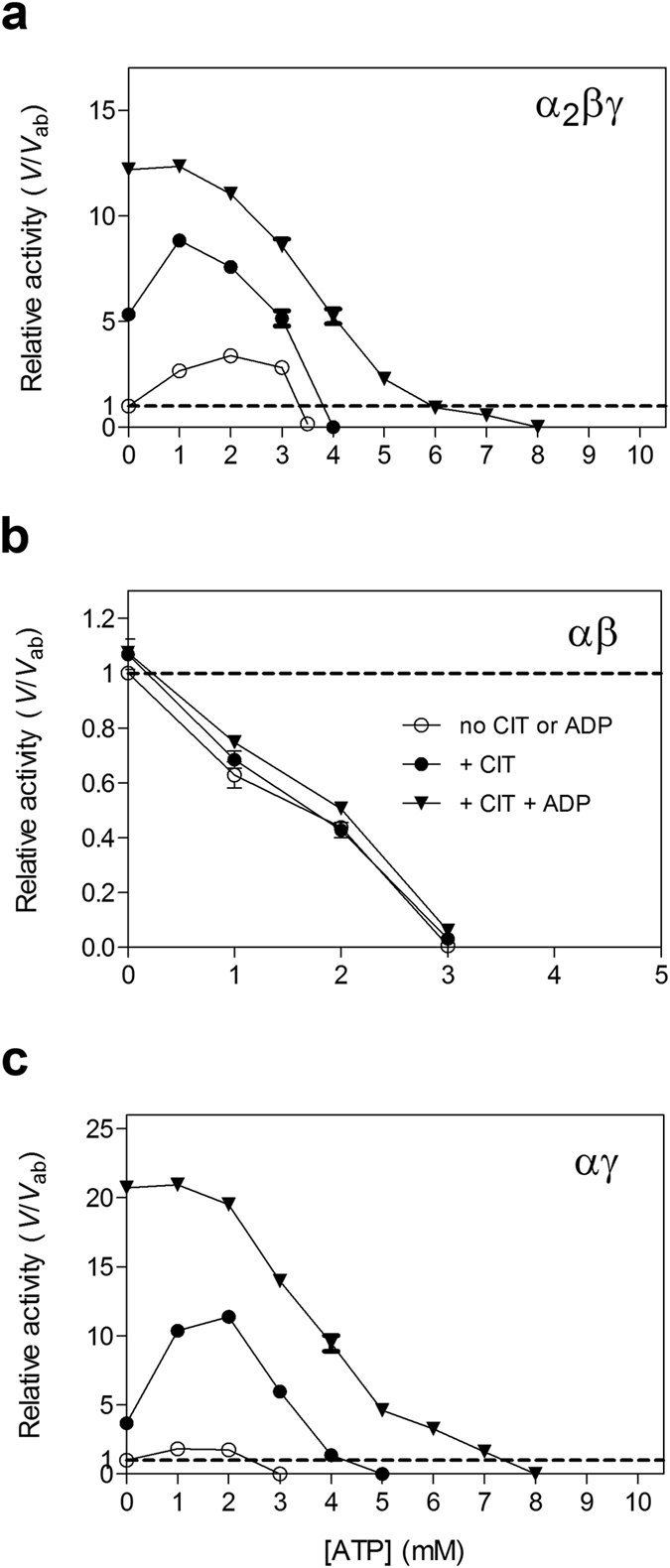
Activation and inhibition effects of ATP. (**a**) The relative activity of the α_2_βγ enzyme *vs.* the concentration of ATP in the absence or presence of positive regulator(s). (**b**) The relative activity of the αβ enzyme *vs.* the concentration of ATP in the absence or presence of positive regulator(s). (**c**) The relative activity of the αγ enzyme *vs.* the concentration of ATP in the absence or presence of positive regulator(s). The activities in the absence of any regulators (*V*_ab_) are defined as 1 and indicated by dashed lines. The activities were measured at the standard conditions with a subsaturating concentration of ICT (0.6 mM for the α_2_βγ and αγ enzymes and 2 mM for the αβ enzyme) in the absence or presence of 1 mM CIT or/and 1 mM ADP and varied concentration of ATP (0–10 mM).

The previous biochemical data showed that the activities of mammalian NAD-IDHs can be negatively regulated by ATP^1^,^11^,^20^. To investigate the regulatory effect of ATP, we measured the activities of the α_2_βγ, αβ and αγ enzymes at different concentrations of ATP in the absence or presence of CIT or both CIT and ADP. Surprisingly, our results show that ATP has both activation and inhibition effects on the activities of the α_2_βγ and αγ enzymes but only inhibition effect on the αβ enzyme (Fig. 3). For the α_2_βγ enzyme, ATP exhibits activation effect at low concentration with a maximum of 2.5-fold increase of the activity at [ATP] of 2 mM in the absence of any regulators, but exhibits inhibition effect at high concentration with no measurable activity at [ATP] of >3.5 mM (Fig. 3a). In the presence of CIT, the dependency of the activity on the ATP concentration displays a similar pattern with a maximum of 9-fold increase of the activity at [ATP] of 1 mM and a complete inhibition at [ATP] of >4 mM. In the presence of CIT and ADP, the α_2_βγ enzyme has been fully activated, and thus ATP exhibits no activation effect but only inhibition effect on the activity at [ATP] of >1 mM with a complete inhibition at [ATP] of >8 mM. Similar to the α_2_βγ enzyme, the activity of the αγ enzyme is slightly activated in the absence of CIT and significantly activated in the presence of CIT by low concentration of ATP, but is inhibited by high concentration of ATP; and additionally ATP exhibits only inhibition effect at [ATP] of >1 mM in the presence of CIT and ADP (Fig. 3c). In contrast, the activity of the αβ enzyme is completely inhibited by ATP even at low concentration in either absence or presence of CIT or CIT and ADP (Fig. 3b).

As the α_2_βγ and αγ enzymes display higher activities in the presence of low ATP concentration, we further analyzed the effects of ATP on the kinetic parameters of these enzymes in the presence of ATP or CIT and ATP (Table 4). Compared to those in the absence of any regulators, the *V*_max,ICT_ of the α_2_βγ enzyme is not significantly affected by ATP or CIT and ATP, but the *S*_0.5,ICT_ is slightly decreased by 2.8-fold in the presence of ATP and substantially decreased by 12.2-fold in the presence of CIT and ATP. Similarly, the *V*_max,ICT_ of the αγ enzyme is also not significantly affected by ATP or CIT and ATP, but the *S*_0.5,ICT_ is decreased by 1.5-fold in the presence of ATP and by 14.5-fold in the presence of CIT and ATP. Consistently, the α_2_βγ enzyme exhibits positive cooperativity for ICT binding with a Hill coefficient of 1.8 and 1.4 in the presence of ATP and both CIT and ATP, respectively; and the αγ enzyme exhibits positive cooperativity with a Hill coefficient of 1.4 in the presence of ATP but no cooperativity in the presence of both CIT and ATP (Table 4). To avoid that the activation effect of ATP is due to contamination of ADP or hydrolyzation of ATP into ADP, we used the nonhydrolyzable ATP analogue AMP-PNP to perform the same assays, and similar activation and inhibition effects are observed (Supplementary Table S2 and Figure S1). These results indicate that ATP can activate the α_2_βγ and αγ enzymes at low concentration but inhibit their activities at high concentration, whereas ATP exhibits only inhibition effect on the αβ enzyme. The activation of the α_2_βγ and αγ enzymes by low concentration of ATP is in a similar manner as ADP but with a slightly weaker effect.

### The γ subunit regulates both the αβ and αγ heterodimers in the holoenzyme

Our biochemical data show that the full activity of the α_2_βγ heterotetramer requires the assembly and cooperative functions of the αβ and αγ heterodimers in both the absence and presence of activators. The previous biochemical data showed that mutation of α-Tyr126 to Phe, Ser or Glu completely abolishes the activity of the α_2_βγ holoenzyme^23^. To investigate the functional roles of the β and γ subunits and the αβ and αγ heterodimers in the α_2_βγ heterotetramer, we introduced the α-Y126F mutation into the αβ and αγ heterodimers separately, and expressed and purified the mutant α_Y126F_β and α_Y126F_γ heterodimers. The mutant α_Y126F_βαγ heterotetramer was assembled by mixing the purified α_Y126F_β and αγ proteins with 1:1 molar ratio, and the mutant αβα_Y126F_γ heterotetramer was assembled by mixing the purified αβ and α_Y126F_γ proteins with 1:1 molar ratio followed by purification using gel filtration. SEC-MALS analyses indicate that introduction of the α-Y126F mutation does not affect the oligomeric states of the αβ, αγ and α_2_βγ proteins in solution: like the wild-type proteins, the mutant α_Y126F_βαγ and αβα_Y126F_γ proteins exist as heterooctamers, and the mutant α_Y126F_β and α_Y126F_γ proteins exist as heterodimers at low concentration but as dimers of heterodimers at high concentration (Supplementary Figure S2). As expected, the mutant α_Y126F_β and α_Y126F_γ heterodimers and the mutant α_Y126F_βα_Y126F_γ heterotetramer have no detectable activity (Table 5). Intriguingly, the mutant α_Y126F_βαγ and αβα_Y126F_γ heterotetramers exhibit comparable *S*_0.5,ICT_ and about half of the catalytic efficiency compared with the wild-type holoenzyme in both the absence and presence of CIT and ADP, indicating that both αβ and αγ heterodimers in the α_2_βγ heterotetramer can be activated by the activators and contribute equally to the full activity of the holoenzyme. On the other hand, our kinetic data show that only the αγ heterodimer alone can be activated by the activators whereas the αβ heterodimer alone cannot. These results led us to speculate that both αβ and αγ heterodimers in the holoenzyme might be regulated by the γ subunit. Meanwhile, our structural and biochemical studies show that the positive regulation of the αγ heterodimer by CIT and ADP is through their binding to the allosteric site in the γ subunit which causes conformational changes at both the allosteric site and the active site^39^. The γ-K151A mutation disrupts the structural communication between the allosteric site and the active site and thus abolishes the activation of the αγ enzyme by CIT and ADP^39^. Thus, we prepared the mutant αβαγ_K151A_ heterotetramer and carried out kinetic study. Indeed, this mutant holoenzyme has very low activity and cannot be activated by CIT and ADP (Table 5). In addition, our kinetic data show that the mutant α_Y126F_βαγ and αβα_Y126F_γ holoenzymes exhibit a Hill coefficient of 1.6–1.7 in the absence of the activators and a Hill coefficient of 1.3–1.4 in the presence of the activators, indicating that there is a cooperative binding for ICT in the absence and presence of the activators. However, the mutant αβαγ_K151A_ holoenzyme exhibits a Hill coefficient of 1.0 in both the absence and presence of the activators, indicating that there is no cooperative binding for ICT. These results together indicate that the γ subunit plays a critical role in the allosteric regulation of the holoenzyme and can regulate both the αβ and αγ heterodimers in the α_2_βγ heterotetramer.

## Discussion

Human NAD-IDH is an allosteric enzyme consisting of the αβ and αγ heterodimers that are assembled into the α_2_βγ heterotetramer and further into the heterooctamer. The previous biochemical studies showed that the α subunit plays the catalytic role and the β and γ subunits the regulatory roles^19^,^22^,^23^,^24^,^25^. However, the enzymatic properties of the composing αβ and αγ heterodimers are not well studied and the exact functional roles of the β and γ subunits in the α_2_βγ heterotetramer are still elusive. In this work, we systematically characterized the enzymatic properties of the α_2_βγ heterotetramer and the αβ and αγ heterodimers in the absence and presence of different regulators, which reveal new mechanistic insights into the function and allosteric regulation of human NAD-IDH.

Our biochemical data show that the α_2_βγ enzyme has basal activity in the absence of any regulators, and can be slightly activated by CIT or ADP alone but substantially activated by CIT and ADP together, indicating that the two activators work synergistically. The binding of CIT or/and ADP enhances the catalytic efficiency of the enzyme by decreasing the *S*_0.5,ICT_ but has no significant effect on the *V*_max,ICT_. In addition, the α_2_βγ enzyme exhibits cooperative binding for ICT in both the absence and presence of CIT or/and ADP. These data indicate that the α_2_βγ enzyme contains at least two ICT-binding sites with positive cooperativity, and CIT and ADP have distinct binding sites and can bind to the enzyme independently and simultaneously in a synergistic manner.

The αγ heterodimer alone exhibits similar kinetic properties as the α_2_βγ enzyme in both the absence and presence of the activator(s) and can also be activated by CIT or/and ADP in similar manners. In the presence of CIT and ADP, the *S*_0.5,ICT_ of the αγ enzyme is decreased by 24.7-fold and the catalytic efficiency is increased by 44.8-fold which is elevated from 19.0% (in the absence of activators) to 55.6% of that of the α_2_βγ enzyme. Moreover, our kinetic data show that the αγ enzyme exhibits cooperative binding for ICT in the absence of any activators and in the presence of ADP, but no cooperative binding in the presence of CIT or both CIT and ADP, suggesting that the αγ enzyme contains two ICT-binding sites probably with one each in the α and γ subunits; CIT binds to one ICT-binding site most likely in the γ subunit and the ADP binding does not occlude the binding of ICT or CIT to this site. These results are consistent with and supported by our structural and biochemical data showing that the αγ heterodimer contains the allosteric site in the γ subunit which is consisted of a CIT-binding site and an ADP-binding site, and the binding of CIT (and ADP) causes conformational changes at the allosteric site which are transmitted to the active site in the α subunit through a cascade of conformational changes at the heterodimer interface, leading to stabilization of the ICT binding at the active site and thus activation of the enzyme^39^. In addition, our mutagenesis data show that mutation of a key residue in the γ subunit (γ-K151A) completely abolishes the activation effect of the holoenzyme. These data together indicate that the γ subunit plays the regulatory role in the αγ heterodimer and α_2_βγ holoenzyme.

In contrast, the αβ enzyme exhibits kinetic properties dissimilar to the αγ and α_2_βγ enzymes and has significantly higher *S*_0.5_ values for ICT and Mn^2 + ^in both the absence and presence of the activator(s). The αβ enzyme cannot be activated by CIT or/and ADP, and the catalytic efficiency is about 9.5% of that of the α_2_βγ enzyme at high concentration of Mn^2 + ^in the absence of the activator(s) and about 0.7% of that of the α_2_βγ enzyme in the presence of CIT and ADP. In addition, the αβ enzyme exhibits no cooperative binding for ICT in both the absence and presence of the activator(s). These data suggest that the αβ enzyme contains only one ICT-binding site most likely in the α subunit and no allosteric site in the β subunit. These results are also consistent with and supported by our structural and biochemical data showing that the αβ enzyme contains no allosteric site in the β subunit and cannot bind CIT or/and ADP due to structural and conformational differences at the “pseudo” allosteric site (Ma *et al*., unpublished data). Nevertheless, our mutagenesis data with the holoenzyme show that the αβ heterodimer also contributes to the full activity of the holoenzyme in both the absence and presence of the activators, and can be activated by CIT and ADP through the γ subunit. These data together suggest that the β subunit plays a structural role in the α_2_βγ heterotetramer to facilitate the assembly and thus ensure the full activity of the holoenzyme. The previous biochemical data showed that several residues of the β subunit, including β-Arg99, β-Tyr137, β-Asp192 and β-Asp217, play important roles in the function and regulation of the α_2_βγ enzyme^19^,^23^,^24^,^25^. Our mutagenesis and biochemical data show that although these mutations have no effect on the basal activity of the αβ enzyme, some of them at the heterodimer interface have some effects on the activity of the α_2_βγ enzyme in the absence and particularly in the presence of the activators (Ma *et al*., unpublished data). It is very likely that the β subunit is involved in the heterodimer-heterodimer and/or heterotetramer-heterotetramer interfaces in the holoenzyme, and the conformational changes at the allosteric site in the γ subunit induced by the binding of activators are transmitted to the active sites in the αγ and αβ heterodimers via the interfaces, and thus mutations of the β subunit at the heterodimer interface would affect the cooperative function(s) of the two heterodimers and/or heterotetramers and hence compromise the full activity of the holoenzyme.

Surprisingly, our biochemical data show that ATP has both activation and inhibition effects on the α_2_βγ and αγ enzymes dependent on the concentration but only inhibition effect on the αβ enzyme, which are different from the previous biochemical data showing that ATP only inhibits the activity of bovine heart NAD-IDH^11^. In the absence of any activators or the presence of CIT, ATP can activate the α_2_βγ and αγ enzymes at low concentration but inhibit their activities at high concentration. In the presence of both CIT and ADP, ATP exhibits only inhibition effect on the activities as the enzymes have been fully activated. Moreover, our kinetic data show that the activation effect of ATP is exerted in a similar manner as ADP. These results lead us to speculate that there might be two different binding sites for ATP in the α_2_βγ and αγ enzymes. One binding site might be the same site for ADP in the γ subunit and the binding of ATP to this site could positively regulate the activity in a synergistic manner with CIT. Indeed, our preliminary structural data show that ATP can bind to the ADP-binding site of the αγ heterodimer in the presence of CIT, which can induce similar conformational changes as the binding of CIT and ADP (Ma *et al*., unpublished data). The other binding site might be at the active site, as suggested earlier^11^, and the binding of ATP to this site would compete with the NAD binding and thus inhibits the activity. It is also possible that high concentration of ATP may compete for binding with the metal ion and thus confers inhibition on the activity. More biochemical and structural studies are needed to dissect the molecular mechanism of the dual effect of ATP.

Taken together, our biochemical data indicate that the αβ and αγ enzymes alone have considerable basal activities but exhibit different enzymatic properties: the αγ enzyme shares very similar kinetic characteristics and allosteric regulation patterns as the α_2_βγ enzyme, whereas the αβ enzyme does not. Both the αβ and αγ heterodimers in the α_2_βγ heterotetramer contribute equally to the full activity of the holoenzyme in both the absence and presence of the activators, and the positive regulation of the αβ and αγ heterodimers in the α_2_βγ heterotetramer is through the γ subunit but not the β subunit. These results together demonstrate that the α subunits play the catalytic role, the γ subunit the regulatory role, and the β subunit the structural role in the α_2_βγ heterotetramer. Further biochemical and structural studies of the α_2_βγ heterotetramer will provide more insights into the molecular basis of the specific functional roles of the β and γ subunits and the αβ and αγ heterodimers in the α_2_βγ heterotetramer and reveal the catalytic and regulatory mechanisms of the α_2_βγ heterotetramer.

## Methods

### Cloning, expression, and purification

The αβ and αγ heterodimers and the α_2_βγ heterotetramer of human NAD-IDH were prepared using a method different from that described previously^22^. Human *IDH3A, IDH3B* (isoform 2), and *IDH3G* genes in vector pReceiver-B01/B02 were purchased from FulenGen (China). The N-terminal 27, 34 and 39 residues of the α, β and γ subunits, respectively, which are the signal peptides for their translocation into the mitochondria^40^, were removed during construction. The DNA fragments of the α, β and γ subunits were individually cloned into the co-expression vector pQlinkN with the C-terminals of the β and γ subunits attached with or without a TEV protease cleavage site and a His_6_ tag, which were then used to construct the pQlinkN-α-β-tev-His_6_, pQlinkN-α-γ-tev-His_6_, pQlinkN-α-β, and pQlinkN-α-γ plasmids, and subsequently the pQlinkN-α-γ-α-β-tev-His_6_ plasmid, following the pQlink cloning procedure^41^.

The pQlinkN-α-β-tev-His_6_ and pQlinkN-α-γ-tev-His_6_ plasmids were transformed into *E. coli* BL21(DE3) Codon-Plus strain (Novagen) for expressions of the αβ and αγ heterodimers, and the pQlinkN-α-γ-α-β-tev-His_6_ plasmid for expression of the α_2_βγ heterotetramer. When the culture of the transformed cells reached an OD_600_ of 0.4~0.6, the protein expression was induced by 0.4 mM IPTG for 20 hr at 23 °C. The bacterial cells were harvested, resuspended, and sonicated on ice in a lysis buffer (50 mM HEPES-Na, pH 7.4, 200 mM NaCl, 0.2 mM MnCl_2_, 10% (w/v) glycerol, and 7.2 mM β-ME) supplemented with 1 mM PMSF. The target protein was firstly purified by affinity chromatography using a Ni-NTA column (Qiagen) with the lysis buffer supplemented with 20 mM and 200 mM imidazole serving as the washing buffer and elution buffer, respectively. The elution fraction was dialyzed against the lysis buffer overnight to lower the concentration of imidazole to < 10 mM; meanwhile, the His_6_-tag was cleaved by TEV protease. The protein mixture was reloaded on a Ni-NTA column, which was then washed with the lysis buffer supplemented with 10 mM imidazole. The flow-through fraction containing the target protein was concentrated by centrifugation using an Amicon Ultra-4 centrifugal filter unit with Ultracel-30 membrane (Millipore), and then purified by gel filtration using a Superdex 200 10/300 GL column (GE Healthcare) equilibrated with the storage buffer (10 mM HEPES, pH 7.4, 200 mM NaCl, and 5 mM β-ME). As the yield of the co-expressed α_2_βγ heterotetramer was much lower than those of the αβ and αγ heterodimers, we assembled the α_2_βγ heterotetramer by either co-purifying the separately expressed αβ and αγ heterodimers using a combination of affinity chromatography and gel filtration, or mixing the purified αβ and αγ heterodimers at 1:1 molar ratio overnight followed by purification using gel filtration chromatography. Both methods produced the α_2_βγ heterotetramer with much higher yields. Purities of the protein samples were assessed by 12% SDS-PAGE with Coomassie blue staining. Concentrations of the proteins were determined using the BCA Protein Assay Kit (Thermo Scientific). Mutants of the αβ and αγ heterodimers containing point mutations in the α, β and γ subunits were constructed using the QuikChange® Site-Directed Mutagenesis kit (Strategene). Expression and purification of the mutants were carried out the same as for the wild-type proteins.

### SEC-MALS analysis

The purity, molecular mass and size distribution of the proteins were analyzed by an analytical light scattering instrument (SEC-MALS) consisting of an Agilent 1260 Infinity Isocratic Liquid Chromatography System, a Wyatt Dawn Heleos II Multi-Angle Light Scattering Detector (Wyatt Technology) and a Wyatt Optilab T-rEX Refractive Index Detector (Wyatt Technology). Analytical size exclusion chromatography was performed at room temperature using a Superdex 200 10/300 GL column (GE Healthcare) equilibrated with a mobile phase containing 10 mM HEPES (pH 7.4), 200 mM NaCl, and 5 mM β-ME. 100 μl protein sample at concentration of 2 mg/ml or 12 mg/ml was injected into the column and eluted at a flow rate of 0.4 ml/min. The column effluent was monitored in-line with three detectors that simultaneously monitor the UV absorption, light scattering and refractive index. The measurements were analyzed using the ASTRA software (Wyatt Technology) to determine the molecular mass of the protein^42^.

### Enzymatic activity assay

The activities of the α_2_βγ, αβ and αγ enzymes were determined by monitoring the time-dependent formation of NADH at 340 nm (ε = 6220 M^−1^ cm^−1^) using a Coulter DU 800 Spectrophotometer (Beckman) at 25 °C. The standard reaction solution (1 ml) consisted of 33 mM Tris-acetate (pH 7.4), 2 ng/ml enzyme, 80 mM _DL_-isocitrate, 2 mM MnCl_2_, and 3.2 mM NAD. The catalytic reaction was initiated by addition of NAD. The specific activity is defined as the amount of NADH produced per minute per milligram of enzyme (μmol/min/mg).

The kinetic parameters of the enzymes in the absence of regulators were determined at the standard conditions with varied concentrations of ICT (0–80 mM), Mn^2+^ (0–2 mM), or NAD (0–3.2 mM) to obtain the *V*_max_ and the *S*_0.5_ for ICT, Mn^2+^, or NAD, respectively. As the αβ enzyme had low activity and very high *S*_0.5,Mn_ at the standard conditions, its kinetic parameters were measured at the conditions with substantially increased concentration of MnCl_2_ (50 mM). The kinetic parameters in the presence of regulators were determined at the standard conditions containing 1 mM CIT or 1 mM ADP (ATP), or both. The production of NADH follows pseudo-first-order kinetics and the time course of the product appearance is well modeled by a linear function up to the time when about 10% of the total substrate was converted to the product. The initial rate was determined from the slope of a linear fit of the early time point data. The kinetic parameters were obtained by fitting the experimental data into the non-Michaelis-Menten equation “*V* = *V*_max_*[S]^h/(*S*_0.5_^h + [S]^h)” using the program Graphpad Prism (Graphpad Software), where “h” is the Hill coefficient, “*S*_0.5_” is the apparent *K*_m_ (the substrate concentration at the reaction velocity of 0.5*V_max_), and “[S]” is the concentration of ICT, Mn^2 + ^, or NAD. All the experiments were performed in three independent measurements and the values were the averages of the three measurements with the standard errors.

For determination of the effect of ATP, the activities of the enzymes were measured at the standard conditions except for a subsaturating concentration of ICT (0.6 mM for the α_2_βγ and αγ enzymes and 2 mM for the αβ enzyme) and varied concentrations of ATP (0–10 mM).

## Additional Information

**How to cite this article:** Ma, T. *et al*. The β and γ subunits play distinct functional roles in the α_2_βγ heterotetramer of human NAD-dependent isocitrate dehydrogenase. *Sci. Rep.* **7**, 41882; doi: 10.1038/srep41882 (2017).

**Publisher's note:** Springer Nature remains neutral with regard to jurisdictional claims in published maps and institutional affiliations.

## Supplementary Material

Supplementary Information

## Figures and Tables

**Table 1:** Specific activities of the α_2_βγ, αβ and αγ enzymes.

Enzyme	Specific activity (*μmol/min/mg*)			
	This work	Ehrlich *et al*.^2^	Soundar *et al*.^19^	Kim *et al*.^22^
α_2_βγ	20.2 ± 0.3	15–20	21.7	0.2359 ± 0.0320
αβ	3.33 ± 0.13	2.9		0.0046 ± 0.0002
αγ	7.27 ± 0.31	7.8		0.0255 ± 0.0007

The specific activities of the enzymes were measured at the standard conditions: 33 mM Tris-acetate, pH 7.4, 80 mM ICT, 2 mM MnCl_2_, and 3.2 mM NAD.

**Table 2:** Kinetic parameters of the α_2_βγ, αβ and αγ enzymes in the absence of any regulators.

Enzyme	*V*_max,ICT_	*S*_0.5,ICT_	Hill coefficient for ICT	*S*_0.5,Mn_	*S*_0.5,NAD_	*k*_*cat*_^a^	*k*_*cat*_*/S*_0.5,ICT_
	*μmol/mg/min*	*mM*		*μM*	*μM*	*s*^*−1*^	*s*^*−1*^*mM*^*−1*^
α_2_βγ	20.0 ± 0.1	2.35 ± 0.05	2.0 ± 0.1	60.2 ± 6.0	143 ± 5	26.7 ± 0.1	11.36 ± 0.04
αβ	10.9 ± 0.3	13.4 ± 0.1	1.1 ± 0.0	5305 ± 314	326 ± 15	14.6 ± 0.4	1.08 ± 0.03
αγ	7.29 ± 0.11	4.49 ± 0.15	2.0 ± 0.1	95.1 ± 3.2	238 ± 18	9.72 ± 0.15	2.16 ± 0.03

The *V*_max_ and *S*_0.5_ of the α_2_βγ and αγ enzymes were determined at the standard conditions with varied concentrations of ICT, or MnCl_2_, or NAD. The *V*_max_ and *S*_0.5_ of the αβ enzyme were determined at the same conditions but with higher concentration of MnCl_2_ (50 mM).

^a^A molecular mass of 80 kDa was used to calculate the mole of enzyme in heterodimeric form per mg of protein (1.25 × 10^−8^ mol of dimeric enzyme/mg of protein).

**Table 3:** Kinetic parameters of the α_2_βγ, αβ and αγ enzymes in the presence of positive regulators.

Enzyme	+CIT			+ADP			+CIT+ADP				
	*V*_max,ICT_	*S*_0.5,ICT_	Hill coefficient for ICT	*V*_max, ICT_	*S*_0.5, ICT_	Hill coefficient for ICT	*V*_*max,ICT*_	*S*_0.5,ICT_	Hill coefficient for ICT	*k*_*cat*_^a^	*k*_*cat*_*/S*_0.5,ICT_
	*μmol/mg/min*	*mM*		*μmol/mg/min*	*mM*		*μmol/mg/min*	*mM*		*s*^*−1*^	*s*^*−1*^*mM*^*−1*^
α_2_βγ	20.7 ± 1.3	1.27 ± 0.06	1.5 ± 0.1	22.1 ± 0.3	0.868 ± 0.021	2.0 ± 0.1	21.3 ± 0.4	0.163 ± 0.007	1.5 ± 0.1	28.4 ± 0.5	174 ± 3
αβ	11.2 ± 0.4	12.1 ± 1.1	1.1 ± 0.1	11.2 ± 0.3	12.1 ± 1.1	1.0 ± 0.1	11.9 ± 0.3	12.6 ± 0.8	1.2 ± 0.1	15.8 ± 0.4	1.25 ± 0.03
αγ	10.0 ± 0.2	2.61 ± 0.12	1.2 ± 0.0	9.42 ± 0.09	1.69 ± 0.05	1.6 ± 0.1	13.1 ± 0.4	0.182 ± 0.015	1.0 ± 0.0	17.6 ± 0.5	96.7 ± 2.7

The *V*_max,ICT_ and *S*_0.5,ICT_ of the α_2_βγ and αγ enzymes in the presence of 1 mM CIT or 1 mM ADP or both were determined at the standard conditions with varied concentrations of ICT. The *V*_max,ICT_ and *S*_0.5,ICT_ of the αβ enzyme were determined at the same conditions but with higher concentration of MnCl_2_ (50 mM) and varied concentrations of ICT.

^a^A molecular mass of 80 kDa was used to calculate the mole of enzyme in heterodimeric form per mg of protein (1.25 × 10^−8^ mol of dimeric enzyme/mg of protein).

**Table 4:** Kinetic parameters of the α_2_βγ and αγ enzymes in the presence of ATP or both CIT and ATP.

Enzyme	+ATP			+CIT+ATP				
	*V*_max, ICT_	*S*_0.5,ICT_	Hill coefficient for ICT	*V*_max, ICT_	*S*_0.5,ICT_	Hill coefficient for ICT	*k*_*cat*_^a^	*k*_*cat*_*/S*_0.5,ICT_
	*μmol/mg/min*	*mM*		*μmol/mg/min*	*mM*		*s*^*−1*^	*s*^*−1*^*mM*^*−1*^
α_2_βγ	17.7 ± 0.1	0.825 ± 0.021	1.8 ± 0.1	17.6 ± 0.1	0.193 ± 0.005	1.4 ± 0.0	23.4 ± 0.1	121 ± 1
αγ	6.62 ± 0.19	2.98 ± 0.16	1.4 ± 0.1	8.94 ± 0.36	0.309 ± 0.014	1.1 ± 0.0	11.9 ± 0.5	38.5 ± 1.6

The *V*_max,ICT_ and *S*_0.5,ICT_ in the presence of 1 mM ATP or both 1 mM CIT and 1 mM ATP were determined at the standard conditions with varied concentrations of ICT.

^a^A molecular mass of 80 kDa was used to calculate the mole of enzyme in heterodimeric form per mg of protein (1.25 × 10^−8^ mol of dimeric enzyme/mg of protein).

**Table 5:** Kinetic parameters of the mutant holoenzyme.

Enzyme	Specific activity	No activators				CIT+ADP			
		*V*_max, ICT_	*S*_0.5,ICT_	*k*_*cat*_^a^/*S*_0.5,ICT_	Hill coefficient for ICT	*V*_max, ICT_	*S*_0.5,ICT_	*k*_*cat*_/*S*_0.5,ICT_	Hill coefficient for ICT
	*μmol/mg/min*	*μmol/mg/min*	*mM*	*s*^*−1*^ *mM*^*−1*^		*μmol/mg/min*	*mM*	*s*^*−1*^*mM*^*−1*^	
αβαγ	20.2 ± 0.3	20.0 ± 0.1	2.35 ± 0.05	11.36 ± 0.04	2.0 ± 0.1	21.3 ± 0.4	0.163 ± 0.007	174 ± 3	1.5 ± 0.1
α_Y126F_β	0	ND^c^	ND	ND	ND	ND	ND	ND	ND
α_Y126F_γ	0	ND	ND	ND	ND	ND	ND	ND	ND
α_Y126F_βα_Y126F_γ	0	ND	ND	ND	ND	ND	ND	ND	ND
αβα_Y126F_γ	8.45 ± 0.14	8.47 ± 0.42	2.12 ± 0.13	5.32 ± 0.13	1.6 ± 0.1	8.43 ± 0.32	0.148 ± 0.005	75.9 ± 5.1	1.4 ± 0.1
α_Y126F_βαγ	9.00 ± 0.15	9.07 ± 0.39	2.41 ± 0.12	5.02 ± 0.12	1.7 ± 0.1	9.12 ± 0.39	0.129 ± 0.006	94.2 ± 3.0	1.3 ± 0.1
αβαγ_K151A_^b^	1.76 ± 0.11	8.24 ± 0.59	21.9 ± 1.1	0.501 ± 0.035	1.0 ± 0.1	8.02 ± 0.42	17.6 ± 1.5	0.607 ± 0.032	1.0 ± 0.1

The *V*_max,ICT_ and *S*_0.5,ICT_ in the absence or presence of both 1 mM CIT and 1 mM ADP were determined at the standard conditions with varied concentrations of ICT, except for those noted specifically.

^a^A molecular mass of 80 kDa was used to calculate the mole of enzyme in heterodimeric form per mg of protein (1.25 × 10^−8^ mol of dimeric enzyme/mg of protein).

^b^The mutant αβαγ_K151A_ enzyme has the *S*_0.5,Mn_ and *S*_0.5,NAD_ of 5.10 ± 0.46 mM and 1.54 ± 0.27 mM, respectively, which are much higher than those of the wild-type enzyme. Thus, the *V*_max, ICT_ and *S*_0.5,ICT_ were determined at higher concentrations of MnCl_2_ (50 mM) and NAD (10 mM).

^c^ND: not detectable.
