# Supplementary material for: The β and γ subunits play distinct functional roles in the α2βγ heterotetramer of human NAD-dependent isocitrate dehydrogenase
Source: Sci Rep. 2017 Jan 31;7:41882. doi: 10.1038/srep41882 (PMC5282582; doi:10.1038/srep41882)

SUPPLEMENTARY INFORMATION

The β and γ subunits play distinct functional roles in the $\alpha_2\beta\gamma$ heterotetramer of human NAD-dependent isocitrate dehydrogenase

Tengfei Ma[1], Yingjie Peng[1], Wei Huang[1], Yabing Liu[2], and Jianping Ding[1,3,4,5,*]

[1] National Center for Protein Science Shanghai, State Key Laboratory of Molecular Biology, Center for Excellence in Molecular Cell Science, Institute of Biochemistry and Cell Biology, Shanghai Institutes for Biological Sciences, Chinese Academy of Sciences, 320 Yueyang Road, Shanghai 200031, China
[2] School of Life Sciences, Shanghai University, 333 Nanchen Road, Shanghai 200444, China
[3] School of Life Science and Technology, ShanghaiTech University, 100 Haike Road, Shanghai 201210, China
[4] Shanghai Science Research Center, Chinese Academy of Sciences, 333 Haike Road, Shanghai 201210, China
[5] Collaborative Innovation Center for Genetics and Development, Fudan University, 2005 Songhu Road, Shanghai 200438, China
*To whom correspondence should be addressed: Jianping Ding (Phone: +86-21-5492-1619, E-mail: jpding@sibcb.ac.cn)

Table S1. Effects of metal ions on the activities of the $\alpha_2\beta\gamma$, $\alpha\beta$ and $\alpha\gamma$ enzymes.

The activities of the $\alpha_2\beta\gamma$ and $\alpha\gamma$ enzymes were determined at the standard condition with different metal ions, and those of the $\alpha\beta$ enzyme at increased concentrations of DL-isocitrate (80 mM) and metal ion (50 mM). The values are means of three independent measurements with standard errors.

Metal ion	Relative activity (%)		
	$\alpha_2\beta\gamma$	$\alpha\beta$	$\alpha\gamma$
None	< 1	< 1	< 1
Mn^{2+}	100.0±2.8	100.0±1.3	100.0±1.3
Mg^{2+}	61.3±1.2	36.3±1.2	42.9±0.3
Co^{2+}	41.0±0.4	16.6±0.2	21.7±1.2
Zn^{2+}	32.3±1.2	16.7±0.4	14.7±0.6
Ca^{2+}	< 1	< 1	< 1
Ni^{2+}	4.9±0.4	< 1	< 1

Table S2. Kinetic parameters of the $\alpha_2\beta\gamma$ and $\alpha\gamma$ enzymes in the presence of both CIT and AMP-PNP.

The $V_{max,ICT}$ and $S_{0.5,ICT}$ in the presence of both 1 mM CIT and 2 mM AMP-PNP were determined at the standard conditions with varied concentrations of ICT.

Enzyme	+CIT+AMP-PNP				
	$V_{max\ ICT}$	$S_{0.5,ICT}$	Hill coefficient for ICT	$kcat$ [a]	$kcat/S_{0.5,ICT}$
	μmol/mg/min	*mM*		s^{-1}	$s^{-1}mM^{-1}$
$\alpha_2\beta\gamma$	17.9±0.2	0.148±0.004	1.4±0.1	23.8±0.3	161±2
$\alpha\gamma$	10.7±0.3	0.330±0.025	1.1±0.1	14.3±0.4	43.3±1.2

[a] A molecular mass of 80 kDa was used to calculate the mole of enzyme in heterodimeric form per mg of protein (1.25×10^{-8} mol of dimeric enzyme/mg of protein).

Figure S1. Activation and inhibition effects of AMP-PNP.

(a) The relative activity of the $\alpha_2\beta\gamma$ enzyme *vs.* the concentration of AMP-PNP in the absence or presence of positive regulator(s). (b) The relative activity of the $\alpha\beta$ enzyme *vs.* the concentration of AMP-PNP in the absence or presence of positive regulator(s). (c) The relative activity of the $\alpha\gamma$ enzyme *vs.* the concentration of AMP-PNP in the absence or presence of positive regulator(s). The activities in the absence of any regulators (V_{ab}) are defined as 1 and indicated by dashed lines. The activities were measured at the standard conditions with a subsaturating concentration of ICT (0.6 mM for the $\alpha_2\beta\gamma$ and $\alpha\gamma$ enzymes and 2 mM for the $\alpha\beta$ enzyme) in the absence or presence of 1 mM CIT or/and 1 mM ADP and varied concentration of AMP-PNP (0-10 mM).

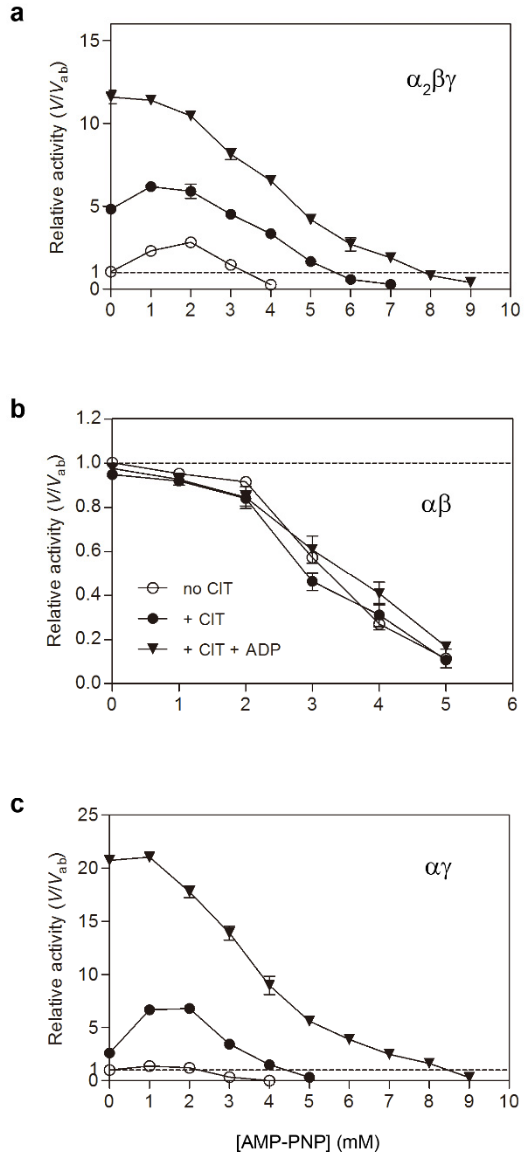

Figure S2. SEC-MALS analyses of the $\alpha_{Y126F}\beta\alpha\gamma$, $\alpha\beta\alpha_{Y126F}\gamma$, $\alpha_{Y126F}\beta$ and $\alpha_{Y126F}\gamma$ proteins of human NAD-IDH.

Chromatograms show the readings from the light scattering (red) at 90°, refractive index (blue), and UV (green) detectors. The left and right vertical axes represent the light scattering detector reading and the molecular mass. The black curve represents the calculated molecular mass. The $\alpha_{Y126F}\beta\alpha\gamma$ protein obtained by mixing the purified $\alpha_{Y126F}\beta$ and $\alpha\gamma$ proteins with 1:1 molar ratio shows an elution peak at about 11 ml corresponding to an average molecular mass of about 288 kDa at the injection protein concentration of 2 mg/ml. The $\alpha\beta\alpha_{Y126F}\gamma$ protein obtained by mixing the purified $\alpha\beta$ and $\alpha_{Y126F}\gamma$ proteins with 1:1 molar ratio shows an elution peak at about 11 ml corresponding to an average molecular mass of about 283 kDa at the injection protein concentration of 1 mg/ml. The $\alpha_{Y126F}\beta$ and $\alpha_{Y126F}\gamma$ proteins show an elution peak at about 14 ml corresponding to an average molecular mass of about 76 kDa at the injection protein concentration of 2 mg/ml, and an elution peak at about 13 ml corresponding to an average molecular mass of about 130 kDa at the injection protein concentration of 12 mg/ml.

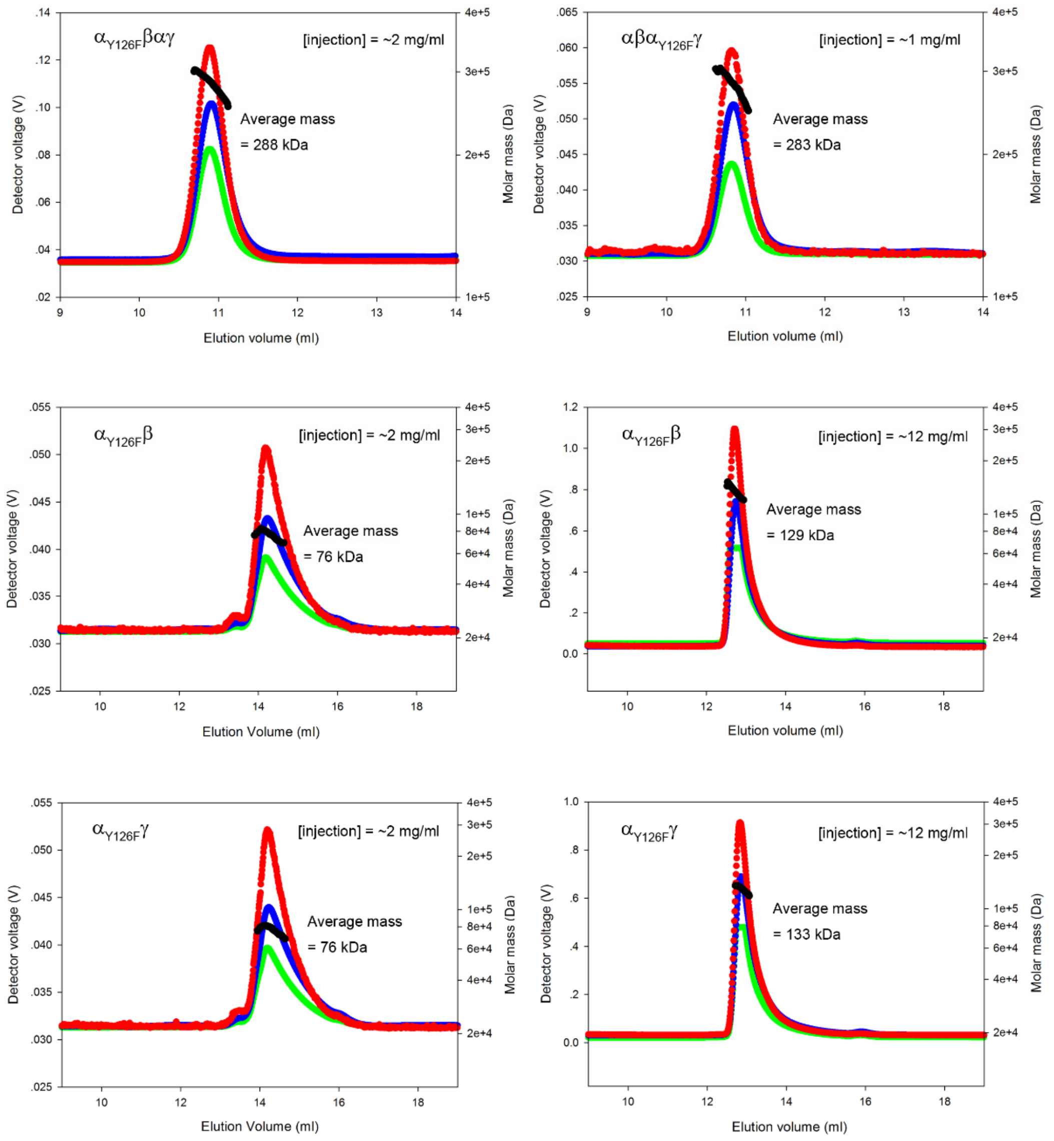

Supplement: Supplementary Information [file srep41882-s1.pdf]
